# Supplementary material for: Resolving the full spectrum of human genome variation using Linked-Reads
Source: Genome Res. 2019 Apr;29(4):635–45. doi: 10.1101/gr.234443.118 (PMC6442396; doi:10.1101/gr.234443.118)
Supplement: Supplemental Material [file supp_gr.234443.118_Supplemental_longranger-2.2.zip › longranger-2.2.tar.bz2!/lr2.2-public/lib/stan/cmdstan/stan/doc/api/html/dims_8hpp_source.html]

Stan: src/stan/math/matrix/dims.hpp Source File


|  |  |
| --- | --- |
|  | Stan  2.5.0  probability, sampling & optimization |


- Main Page
- Related Pages
- Namespaces
- Classes
- Files

- File List
- File Members

All Classes Namespaces Files Functions Variables Typedefs Enumerations Enumerator Friends Macros Pages

- src
- stan
- math
- matrix

dims.hpp

Go to the documentation of this file.

1 #ifndef STAN\_\_MATH\_\_MATRIX\_\_DIMS\_HPP

2 #define STAN\_\_MATH\_\_MATRIX\_\_DIMS\_HPP

3

4 #include <vector>

5 #include <stan/math/matrix/Eigen.hpp>

6

7 namespace stan {

8  namespace math {

9

10  template <typename T>

11  inline

12  void

13  dims(const T& x, std::vector<int>& result) {

14  /\* no op \*/

15  }

16  template <typename T, int R, int C>

17  inline void

18  dims(const Eigen::Matrix<T,R,C>& x,

19  std::vector<int>& result) {

20  result.push\_back(x.rows());

21  result.push\_back(x.cols());

22  }

23  template <typename T>

24  inline void

25  dims(const std::vector<T>& x,

26  std::vector<int>& result) {

27  result.push\_back(x.size());

28  if (x.size() > 0)

29  dims(x[0],result);

30  }

31

32  template <typename T>

33  inline std::vector<int>

34  dims(const T& x) {

35  std::vector<int> result;

36  dims(x,result);

37  return result;

38  }

39

40  }

41 }

42 #endif

stan::math::dims

void dims(const T &x, std::vector< int > &result)

**Definition:** dims.hpp:13

Eigen.hpp


---

[ Stan Home Page ]

*© 2011–2014,
Stan Development Team.*
